# β-Blocker Use and Health Status Among Patients With Heart Failure With Preserved Ejection Fraction

**DOI:** 10.1001/jamanetworkopen.2025.29519

**Published:** 2025-08-28

**Authors:** Mohammad Abdel Jawad, John A. Spertus, Yoon J. Cho, Philip G. Jones, Suzanne V. Arnold

**Affiliations:** 1 University of Missouri Kansas City’s Healthcare Institute for Innovations in Quality, Kansas City; 2 Division of Cardiology, Saint Luke’s Mid America Heart Institute, Kansas City, Missouri

## Abstract

**Importance:**

β-Blockers are widely used for patients with heart failure with preserved ejection fraction (HFpEF). However, their association with health status among this population remains unknown.

**Objective:**

To evaluate the association of β-blocker use with health status among patients with HFpEF.

**Design, Setting, and Participants:**

This cohort study was a secondary analysis of the Treatment of Preserved Cardiac Function Heart Failure with an Aldosterone Antagonist randomized clinical trial, which enrolled 3445 patients with HFpEF (left ventricular ejection fraction [LVEF] ≥45%) to receive spironolactone or placebo. We excluded 1678 patients from Georgia or Russia and 41 with missing baseline Kansas City Cardiomyopathy Questionnaire overall summary score (KCCQ-OS) or β-blocker data, resulting in an analytic cohort of 1726 patients enrolled between August 10, 2006, and January 31, 2012. Statistical analysis was performed from July to November 2024.

**Exposure:**

β-Blocker use.

**Main Outcomes and Measures:**

Heath status was assessed with the KCCQ-OS, a heart failure–specific health status patient-reported outcome measure. The cross-sectional association of β-blocker use and baseline KCCQ-OS was assessed using multivariable linear regression, adjusted for demographic and clinical factors, and testing the interaction of β-blocker × LVEF. A second model examined changes in KCCQ-OS from baseline to 4 months and tested the interaction between β-blocker use and spironolactone to examine whether β-blockers were associated with modification of the health status benefit of spironolactone.

**Results:**

Among 1726 patients with HFpEF (mean [SD] age, 71.6 [9.7] years; 862 women [49.9%]; mean [SD] LVEF, 58.1% [7.7%]), 1356 (78.6%) were receiving β-blockers at baseline. β-Blocker use was not significantly associated with concurrent KCCQ-OS (mean difference, −1.1 points [95% CI, −3.7 to 1.4 points]; *P* = .38). This association did not significantly differ by LVEF, although patients with LVEF of 65% or more who used β-blockers had a numerically lower KCCQ-OS score (mean difference, −6.1 points [95% CI, −12.3 to 0.0 points]) compared with LVEF of 55% to 64% (mean difference, 0.0 points [95% CI, −3.9 to 3.8 points) and LVEF of 45% to 54% (mean difference, −0.2 points [95% CI, −4.3 to 3.8 points]) (*P* = .21 for interaction). In the longitudinal model, β-blockers were not associated with modification of the health status benefits of spironolactone at 4 months (mean difference, 2.9 points [95% CI, −1.0 to 4.9 points] with β-blockers vs 0.1 [95% CI, −3.7 to 3.9 points] without; *P* = .20 for interaction).

**Conclusions and Relevance:**

In this cohort study of patients with HFpEF, β-blocker use was not associated with better or worse baseline health status or the modification of the health status benefits of spironolactone at 4 months. Further research is needed to better understand the association of β-blockers with health status among patients with HFpEF.

## Key Points

**Question:**

Among patients with heart failure with preserved ejection fraction (HFpEF), is β-blocker use associated with health status or modification of the health status benefits of spironolactone?

**Findings:**

In this cohort study of 1726 patients with HFpEF from the TOPCAT randomized clinical trial, β-blocker use had no significant association with concurrent health status or modification of the health status benefits of spironolactone at 4 months.

**Meaning:**

These findings suggest that further study is needed to clarify the association of β-blockers with health status among patients with HFpEF, particularly across the range of LVEF.

## Teaser

This cohort study of participants in the TOPCAT randomized clinical trial evaluates the association of β-blocker use with health status among patients with heart failure with preserved ejection fraction.

## Introduction

Due to common comorbidities and older guidelines,^1^ β-blockers are commonly used to treat patients with heart failure with preserved ejection fraction (HFpEF),^2^ although there are growing concerns about the potential harm of this practice. Patients with HFpEF often exhibit an inadequate heart rate response to exercise,^3^ which can translate to reduced exercise capacity that is further exacerbated by β-blockers.^4^ Recent studies have shown that discontinuing β-blockers among patients with HFpEF can improve functional capacity and reduce natriuretic peptides without other apparent adverse effects.^5,6^ Furthermore, observational data suggest that β-blocker use among patients with HFpEF is associated with increased risk of heart failure hospitalization, particularly among those with higher left ventricular ejection fraction (LVEF).^2,7^

Despite this developing evidence of potential harm with β-blockers, their association with patients’ health status, symptoms, function, and quality of life is unknown. To address this gap in knowledge, we used data from the Treatment of Preserved Cardiac Function Heart Failure with an Aldosterone Antagonist (TOPCAT) trial, which demonstrated improved health status with spironolactone, to examine the association of β-blocker use with heart failure–specific health status among patients with HFpEF.^8^

## Methods

### Trial Design and Patients

The TOPCAT trial was a multinational, randomized clinical trial of spironolactone vs placebo among patients with symptomatic HFpEF (defined as heart failure hospitalization within the prior year or elevated natriuretic peptide level and LVEF ≥45%) conducted between August 10, 2006, and January 31, 2012 (NCT00094302). Patients from Russia and Georgia were excluded because of data validity concerns.^9^ The TOPCAT trial was approved by local institutional review boards, and all patients provided informed consent. This cohort study was deemed exempt by the St Luke’s institutional review board because the data were deidentified. This cohort study followed the Strengthening the Reporting of Observational Studies in Epidemiology (STROBE) reporting guideline.

Health status was assessed with the Kansas City Cardiomyopathy Questionnaire overall summary score (KCCQ-OS), which encompasses the symptoms, physical and social function, and quality of life related to heart failure. Scores ranged from 0 to 100, with higher scores indicating better health status.

### Statistical Analysis

Statistical analysis was performed from July to November 2024. The primary objectives were to evaluate the association between β-blocker use and health status in a cross-sectional analysis and to assess whether β-blockers were associated with modification of the health status benefits of spironolactone at 4 months. To examine patient-level differences between those receiving vs not receiving β-blockers at baseline, the propensity of a patient being receiving a β-blocker was estimated using a nonparsimonious logistic regression model. Although there were differences among individual patient factors, the distribution of propensity scores between the 2 groups overlapped substantially, supporting the ability to compare the 2 groups with covariate adjustment (eFigure 1 in Supplement 1). Baseline characteristics were compared using standardized mean differences, with a standardized mean difference of greater than 0.1 indicating a significant imbalance between the groups.

The cross-sectional association between β-blocker use and baseline KCCQ-OS was assessed using multivariable linear regression, adjusting for demographic and clinical patient characteristics selected a priori based on their potential to influence health status or confound the association between β-blocker use and health status. As prior analyses suggested increased harm with β-blocker use among those with higher LVEF, the interaction of β-blocker use × LVEF was tested. Additional interaction terms were also tested, including β-blocker use with baseline heart rate, atrial fibrillation, prior myocardial infarction, and history of stroke.

A second model examined changes in KCCQ-OS from baseline to 4 months, explicitly testing the interaction between β-blocker use × treatment assignment, to explore whether β-blockers were associated with modification of the health status benefit of spironolactone. This model included only patients whose β-blocker use was unchanged from baseline to 4 months. We selected the 4-month time point based on prior TOPCAT findings, which showed that most of the health status improvement with spironolactone occurred within the first 4 months and was sustained through 12 months. In addition, given that the hypothesized mechanism of β-blockers in HFpEF, through hemodynamic and heart rate effects, is likely to produce relatively immediate changes in health status, we considered the 4-month follow-up appropriate for this analysis. Missing covariate data were minimal (<1% for all variables), and thus no imputation of missing data was performed. Analyses were performed using SAS, version 9.4 (SAS Institute Inc), with a 2-sided *P* < .05 indicating statistical significance.

## Results

Among the 3445 patients in the TOPCAT trial, we excluded 1678 patients from Georgia or Russia and 41 patients with missing baseline KCCQ or β-blocker data (eFigure 2 in Supplement 1). The analytic cohort included 1726 patients (mean [SD] age, 71.6 [9.7] years; 862 women [49.9%] and 864 men [50.1%]), of whom 1356 (78.6%) were receiving β-blockers at baseline. An additional 283 patients were excluded from the longitudinal analysis due to missing 4-month KCCQ-OS data (n = 72) or changing β-blocker status (n = 211). Compared with those not receiving β-blockers, patients receiving β-blockers were more likely to be men, be younger, have lower LVEF, and have higher rates of coronary artery disease, peripheral artery disease, hypertension, prior percutaneous intervention, and myocardial infarction (Table).

**Table.** Baseline Patient Characteristics

Characteristic	Patients, No. (%)			Standardized mean difference
	Overall (N = 1726)	No β-blocker (n = 370)	β-Blocker (n = 1356)	
Age, mean (SD), y	71.6 (9.7)	72.6 (10.1)	71.3 (9.6)	0.14
Sex				
Female	862 (49.9)	208 (56.2)	654 (48.2)	0.16
Male	864 (50.1)	162 (43.8)	702 (51.8)	
BMI, mean (SD)	33.8 (8.2)	33.7 (8.8)	33.9 (8.0)	−0.02
Country				
US	1125 (65.2)	202 (54.6)	923 (68.1)	0.35
Canada	316 (18.3)	70 (18.9)	246 (18.1)	
Brazil	163 (9.4)	63 (17.0)	100 (7.4)	
Argentina	122 (7.1)	35 (9.5)	87 (6.4)	
Heart rate, mean (SD), beats/min	69.0 (11.2)	70.6 (11.9)	68.6 (11.0)	0.17
Ejection fraction, mean (SD), %	58.1 (7.7)	59.2 (7.7)	57.8 (7.7)	0.19
History of CABG	332 (19.3)	40 (10.8)	292 (21.6)	−0.29
History of PCI	336 (19.5)	50 (13.5)	286 (21.1)	−0.20
History of myocardial infarction	352 (20.4)	52 (14.1)	300 (22.1)	−0.21
Peripheral artery disease	203 (11.8)	34 (9.2)	169 (12.5)	−0.11
History of stroke	154 (8.9)	39 (10.5)	115 (8.5)	0.07
Atrial fibrillation	730 (42.3)	150 (40.5)	580 (42.8)	−0.05
Chronic lung disease	288 (16.7)	74 (20.0)	214 (15.8)	0.11
Current smoking	116 (6.7)	27 (7.3)	89 (6.6)	0.03
Diabetes	771 (44.7)	146 (39.5)	625 (46.1)	−0.14
Hypertension	1551 (89.9)	314 (84.9)	1237 (91.3)	−0.20
Spironolactone randomization	869 (50.3)	191 (51.6)	678 (50.0)	0.03
KCCQ-OS, mean (SD)	58.1 (23.4)	58.3 (23.1)	58.1 (23.5)	0.01

Abbreviations: BMI, body mass index (calculated as weight in kilograms divided by height in meters squared); CABG, coronary artery bypass graft surgery; KCCQ-OS, Kansas City Cardiomyopathy Questionnaire overall summary score; PCI, percutaneous coronary intervention.

In cross-sectional analyses, β-blocker use at baseline was not significantly associated with concurrent KCCQ-OS (mean difference, −1.1 points [95% CI, −3.7 to 1.4 points]; *P* = .38). Although the LVEF × β-blocker interaction was not significant, patients with LVEF of 65% or more who used β-blockers had a numerically lower KCCQ-OS score (mean difference, −6.1 points [95% CI, −12.3 to 0.0 points]) compared with LVEF of 55% to 64% (mean difference, 0.0 points [95% CI, −3.9 to 3.8 points]) and LVEF of 45% to 54% (mean difference, −0.2 points [95% CI, −4.3 to 3.8 points]) (*P* = .21 for interaction). Similarly, the interactions between β-blocker use and baseline heart rate, atrial fibrillation, prior myocardial infarction, and history of stroke were not statistically significant.

In the longitudinal model, β-blocker use was not significantly associated with KCCQ-OS at 4 months (mean difference, −2.0 points [95% CI, −4.8 to 0.8 points]; *P* = .16). There was a numerically greater health status improvement with spironolactone in the β-blocker group (mean difference, 2.9 points [95% CI, 1.0-4.9 points]) compared with those not receiving β-blockers (mean difference, 0.1 points [95% CI, −3.7 to 3.9 points]); however, this difference was not statistically significant (*P* = .20 for interaction).

## Discussion

β-Blockers are a key pillar of guideline-directed medical therapy for heart failure and reduced ejection fraction, but their role in HFpEF remains controversial, given a lack of randomized clinical trial data and inconsistent observational data. In this secondary analysis of the TOPCAT trial, more than 75% of patients were receiving β-blockers at randomization. β-Blocker use was not significantly associated with health status at baseline or 4 months, nor was it associated with modification of the health status benefits of spironolactone. In addition, the association between β-blocker use and baseline health status did not differ significantly across subgroups defined by baseline heart rate, atrial fibrillation, prior myocardial infarction, or history of stroke. However, there were a few nonsignificant findings that may warrant further investigation. First, patients with higher LVEF (≥65%) receiving β-blockers had worse health status than patients with LVEF of 55% to 64% or those with LVEF of 45% to 54%. This finding is consistent with a prior study that showed an increased risk of heart failure hospitalization with β-blockers among patients with higher LVEF^2^ but differed from a second study that showed potential benefit of β-blockers across the range of LVEF.^10^ Second, the association of spironolactone with health status was seen predominantly among patients receiving β-blockers, which may indicate that spironolactone can counter some of the negative associations of β-blockers. However, given the sample size of the current study, particularly among patients not receiving β-blockers, these hypothesis-generating findings need to be explored in larger datasets or, ideally, in prospective trials.

### Limitations

There are several limitations to our study, most notably the nonrandomized use of β-blockers. Although we adjusted for a broad set of covariates, residual confounding from unmeasured or unknown factors remains possible. The association of β-blockers with health status among patients with HFpEF is likely complicated and related to many factors, such as the presence of persistent arrhythmias, baseline heart rate, and chronotropic response to exercise, which we could not account for in this study. Second, health status can be affected by a number of clinical and sociodemographic factors that were not captured in this study, which could introduce type II error. Third, we could not account for the duration of, intensity of, or adherence to β-blocker use.

## Conclusions

In this cohort study of patients with HFpEF, β-blocker use was not associated with significantly different concurrent heart failure–specific health status or modification of the health status benefit of spironolactone. However, the study does not definitively rule out an association of β-blockers with health status among patients with HFpEF, and further studies are needed to better understand the association of β-blockers with health status among patients with HFpEF.

## Notes

Supplement 1. **eFigure 1.** Baseline Propensity Score Distribution **eFigure 2.** Study Cohort

Supplement 2.
Data Sharing Statement
