# Supplementary material for: β-Blocker Use and Health Status Among Patients With Heart Failure With Preserved Ejection Fraction
Source: JAMA Netw Open. 2025 Aug 28;8(8):e2529519. doi: 10.1001/jamanetworkopen.2025.29519 (PMC12395312; doi:10.1001/jamanetworkopen.2025.29519)
Supplement: Supplement 1. — eFigure 1. Baseline Propensity Score Distribution eFigure 2. Study Cohort [file jamanetwopen-e2529519-s001.pdf]

Supplementary Online Content

Abdel Jawad M, Spertus JA, Cho YJ, Jones PG, Arnold SV. β-Blocker use and health status among patients with heart failure with preserved ejection fraction. *JAMA Netw Open.* 2025;8(8):e2529519. doi:10.1001/jamanetworkopen.2025.29519

eFigure 1. Baseline Propensity Score Distribution

eFigure 2. Study Cohort

This supplementary material has been provided by the authors to give readers additional information about their work.

© 2025 Abdel Jawad M et al. *JAMA Network Open.*

eFigure 1. Baseline Propensity Score Distribution

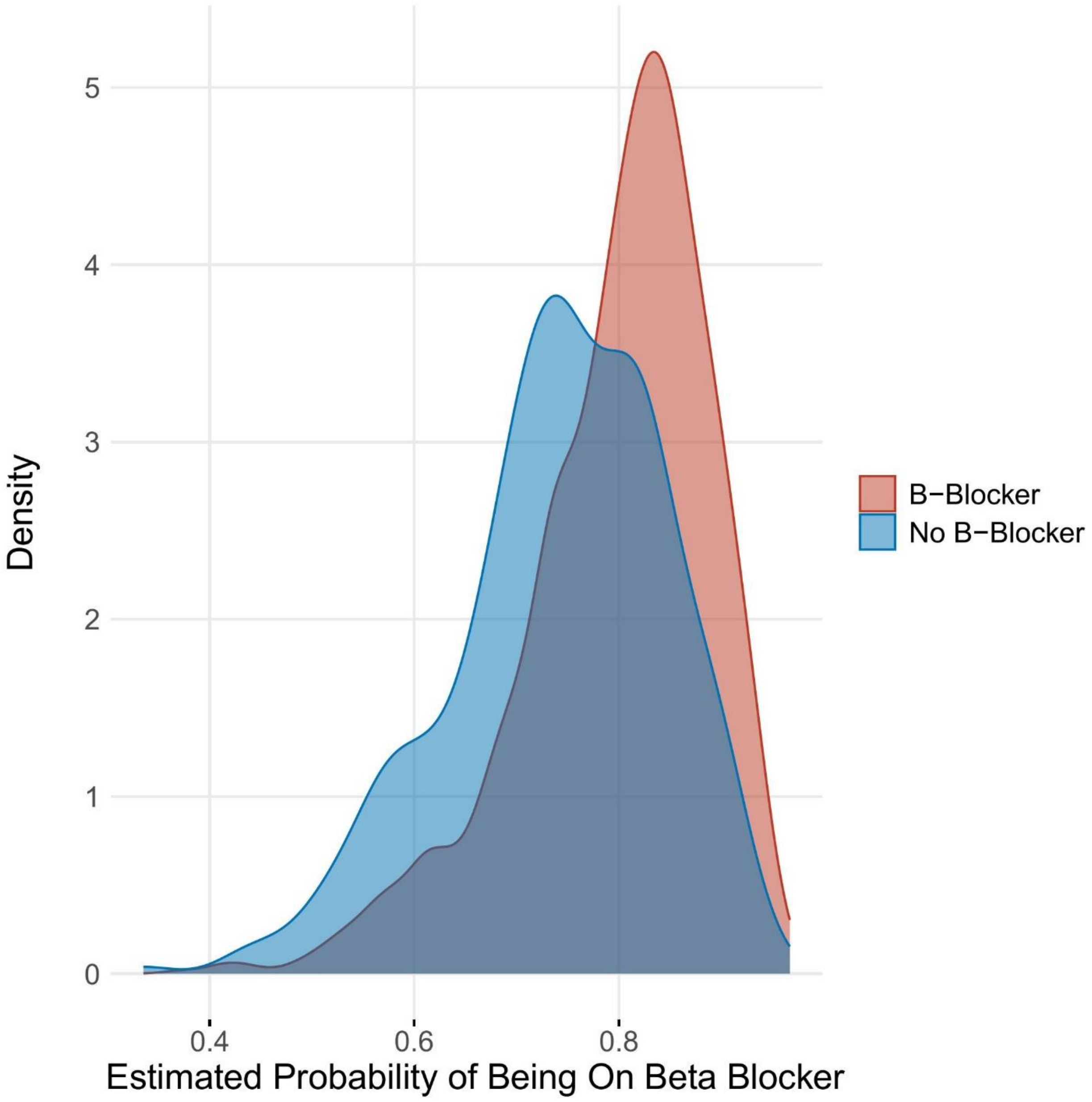

© 2025 Abdel Jawad M et al. *JAMA Network Open.*

eFigure 2. Study Cohort

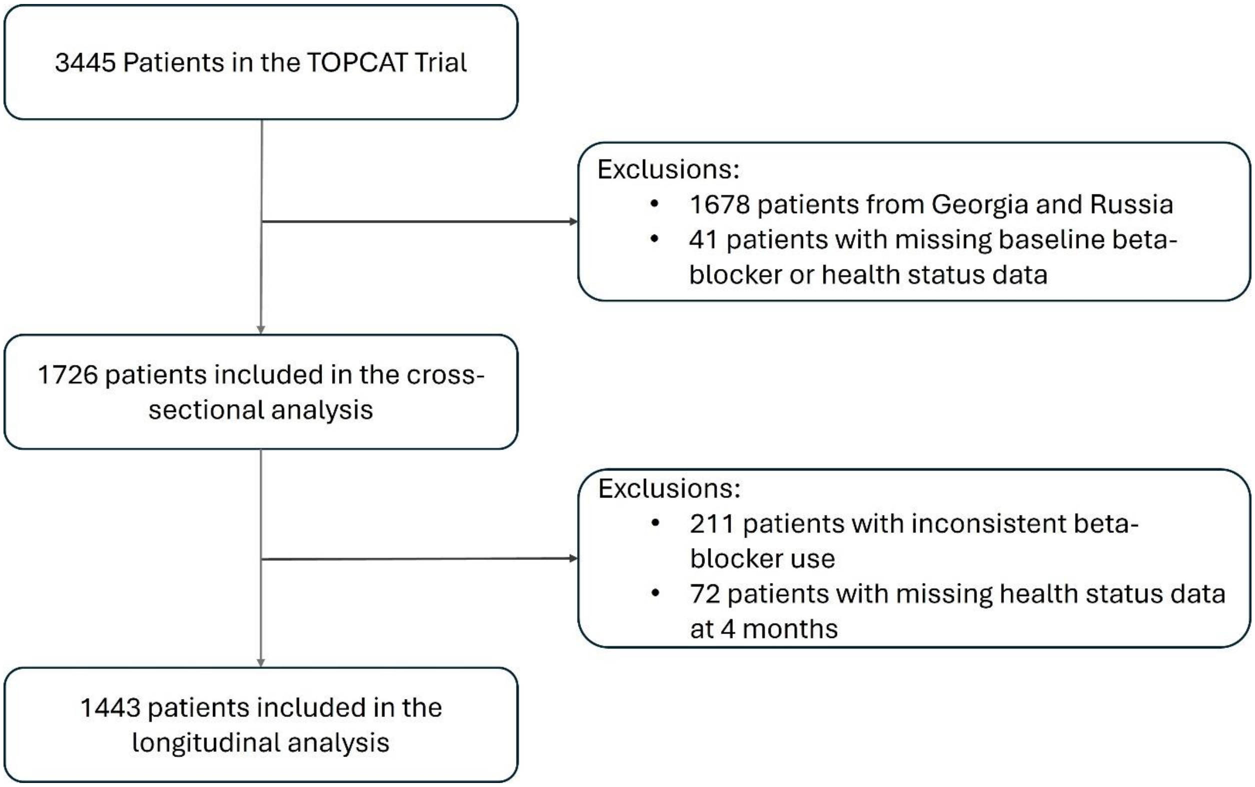

© 2025 Abdel Jawad M et al. *JAMA Network Open.*
