# Supplementary material for: β-Blocker Use and Health Status Among Patients With Heart Failure With Preserved Ejection Fraction
Source: JAMA Netw Open. 2025 Aug 28;8(8):e2529519. doi: 10.1001/jamanetworkopen.2025.29519 (PMC12395312; doi:10.1001/jamanetworkopen.2025.29519)
Supplement: Supplement 2. — Data Sharing Statement [file jamanetwopen-e2529519-s002.pdf]

Data Sharing Statement

Abdel Jawad. β-Blocker Use and Health Status Among Patients With Heart Failure With Preserved Ejection Fraction. *JAMA Netw Open*. Published August 28, 2025. doi:10.1001/jamanetworkopen.2025.29519

Data
Data available: No
